# Cumulative social disadvantage and inequitable utilisation of healthcare and long-term care services among older adults with complex needs: a cross-sectional study in China

**DOI:** 10.7189/jogh.16.04352

**Published:** 2026-10-30

**Authors:** Peng Zhang, Jiabin Xu, Jiajie Xu, Bifan Zhu, Hansheng Ding, Lanshu Zhou, Yunwei Zhang

**Affiliations:** 1 School of Nursing, Shanghai University of Traditional Chinese Medicine, Shanghai, China; 2 Key Laboratory of Geriatric Long-term Care, Naval Medical University, Shanghai, China; 3 Shanghai Health Development Research Center, Shanghai, China

**Keywords:** aged, complex needs, social disadvantages, healthcare services, long-term care services

## Abstract

**Background:**

Older adults with complex needs (CNs) exhibit substantial demands for healthcare and long-term care (LTC) services. Although social disadvantage markedly impairs access to services, empirical research on this population remains sparse, and key dimensions remain unexplored. While LTC services can influence healthcare service utilisation, evidence regarding their role in the association between social disadvantage and healthcare service utilisation among this population remains limited.

**Methods:**

We conducted a cross-sectional study using data from the Long-Term Care Insurance of Shanghai, China, from January to June 2023. We combined five domains of social disadvantage into a cumulative score and categorised it into quintiles. Negative binomial regression models evaluated associations between cumulative social disadvantage (including its quintiles) and its individual dimensions and utilisation of healthcare and LTC services. A parallel multiple mediation analysis examined the mediating roles of formal and informal care between cumulative social disadvantage and healthcare service utilisation.

**Results:**

Among 42,499 CN older adults, increasing cumulative social disadvantage was associated with graded reductions in the utilisation of healthcare and LTC services. Educational disadvantage and healthcare access disadvantage exhibited the strongest negative associations with care service utilisation. We observed significant additive interactions between these two disadvantages for outpatient visit frequency (relative excess risk due to interaction (RERI) = 0.093), hospital stay duration (RERI = 0.576), formal care frequency (RERI = 0.191), and informal care frequency (RERI = 0.379). Although LTC services statistically mediated the association between cumulative social disadvantage and healthcare service utilisation, the proportion mediated remained below 1%.

**Conclusions:**

Cumulative social disadvantage was significantly correlated with reduced utilisation of healthcare and LTC services among CN older adults. Policymakers should pay close attention to the social disadvantages experienced by CN older adults, focusing on healthcare access and educational attainment, while working to increase outpatient visit utilisation. Furthermore, fostering the synergistic development of healthcare and LTC services is essential.

With accelerating global population ageing, the growing population with complex needs (CNs), characterised by three or more chronic conditions and functional impairment [1–4], has emerged as a major public health challenge [5]. This population is heavily concentrated among older adults [6,7]. To address this, a substantial allocation of both healthcare and long-term care (LTC) resources is required [2,8]. Existing literature indicates that while CN populations represent only 5% of the total population, they account for over 50% of healthcare expenditures [1]. Concurrently, they also exhibit a high utilisation rate of LTC services [2,9]. Prominent disparities in healthcare and LTC service utilisation remain particularly pronounced within this population [10]. Therefore, establishing tailored care management is critical to optimise resource allocation and mitigate care inequities in this population.

Existing literature has advanced integrated care models for CN populations, emphasising continuity and coordination of care [7,11–13] and encompassing healthcare and LTC services [14]. While these models primarily target service delivery processes, they often overlook how social disadvantages affect individuals’ ability to access and use these services [10,14]. Importantly, CN populations show pronounced deficits in social determinants of health, resulting in significantly greater social disadvantage [7,10,15]. Consequently, addressing care disparities in CN populations requires moving beyond service delivery processes to incorporate social disadvantage into care management frameworks.

Pinheiro advocates for considering social disadvantage as a prerequisite for mitigating care access inequities among CN populations [16]. Social disadvantage transcends a unidimensional construct, encompassing economic stability, education access and quality, healthcare access, built environment, and social and community context [17]. Due to the overlapping nature of these factors, vulnerable individuals often experience concurrent adversities across several dimensions, resulting in cumulative social disadvantage [18]. However, research examining the impact of cumulative social disadvantage on care utilisation remains limited, as prior studies have primarily focused on isolated domains and healthcare utilisation alone [19–21]. This not only hinders the integration of healthcare and LTC services for CN populations but also limits administrators' ability to mitigate social disadvantages and rectify care service inequities.

The use of LTC services plays a pivotal role in reducing avoidable healthcare service utilisation [22,23]. With the large-scale piloting of LTC insurance in China, managing the interplay between LTC services and healthcare services has become essential [24]. However, when individuals face severe social disadvantages, their ability to access LTC resources is often compromised [25,26]. LTC services include both formal and informal care, which may influence healthcare utilisation differently [27]. However, empirical evidence remains insufficient on whether formal or informal care can reduce avoidable healthcare utilisation among CN older adults facing social disadvantage.

Therefore, we aimed to examine the associations between cumulative social disadvantage and the utilisation of care services among the CN older adult population, with three specific objectives: to examine the gradients in healthcare and LTC service utilisation associated with cumulative social disadvantage; to identify the key dimensions of social disadvantage associated with care service utilisation and examine their synergistic effects; and to examine the mediating roles of LTC services in the association between cumulative social disadvantage and healthcare service utilisation. These findings provide empirical evidence to address disparities in care service utilisation among CN older adults and to optimise integrated care resource allocation.

## METHODS

### Study design and data source

We conducted a cross-sectional study using administrative assessment data obtained from the Shanghai Healthcare Security Bureau. We used this data to determine the care-need levels of individuals covered by LTC insurance. Qualified professionals reviewed and verified all data before uploading them to a database. The development and validation of this questionnaire have been detailed previously [28,29]. Pairs of certified assessors conducted home-based assessments, including a healthcare professional (primarily a general practitioner familiar with the applicant’s health status) and a certified social insurance assessor. This structured, assessor-administered design yielded exceptionally low item non-response rates and ensured high data consistency and accuracy. Complete-case analyses were performed for all data. We followed the STROBE guidelines for observational studies (Checklist S1 in the **Online Supplementary Document**) [30].

### Study participants

We used data collected from CN older adults between January and June 2023. Following Commonwealth Fund criteria, we defined CN status as multimorbidity and functional limitations [1]. Multimorbidity was determined using a list of chronic conditions established by intersecting two authoritative sources (Table S1 in the **Online Supplementary Document**) [7,31], with individuals diagnosed with three or more conditions from this list by general practitioners classified as having multimorbidity. We measured functional impairment using Barthel Index questionnaire items [32,33] and categorised participants as severe (0–8), moderate (9–12), mild (13–19), or independent (20) [32].

We included participants aged ≥60 years, those classified as CN status, and those able to provide valid responses independently or with assistance, with written informed consent (Figure S1 in the **Online Supplementary Document**).

### Measures

#### Social disadvantage

We extracted variables reflecting social disadvantage from standardised questionnaires. Following the Healthy People 2030 framework [34], cumulative social disadvantages were categorised into economic stability, education, healthcare access, built environment, and social and community context (**Figure 1**).

**Figure 1:**
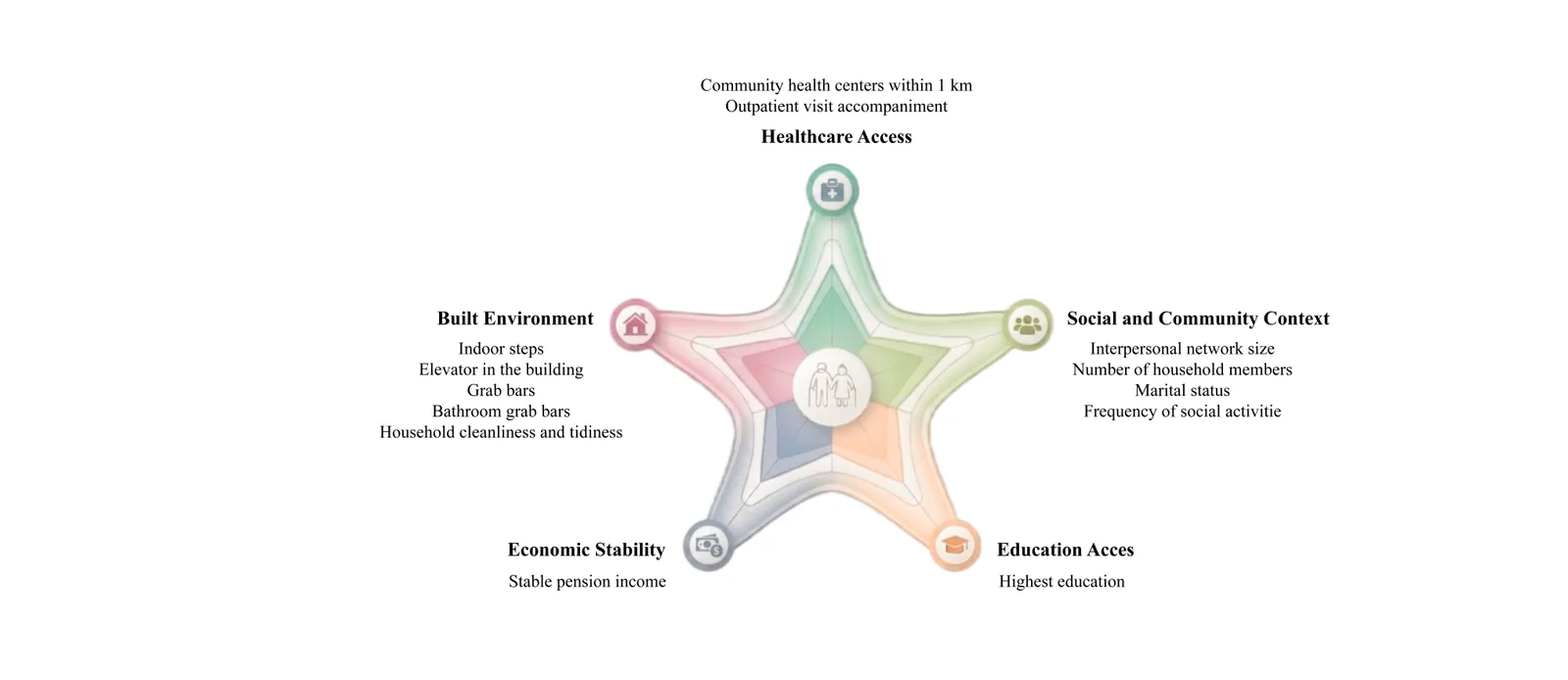
Structural components of the cumulative social disadvantage score. All domains were scored on a scale from 0 to 1. We assigned equal weight to each domain and to each component variable within each domain.

We assessed economic stability based on the presence of a stable pension income, whereas economic instability was characterised by the absence of a pension [35]. We assessed education by the participant’s highest level of educational attainment, classifying illiteracy as a disadvantage [36,37]. Healthcare access was evaluated through primary care availability and access to healthcare services [38,39], determined by whether respondents lived within 1 km of community health centres and received accompaniment to outpatient visits, with the absence of both classified as a disadvantage. Built environment reflected housing quality and environmental conditions through indoor steps, elevators, grab bars, bathroom grab bars, and household cleanliness [40]. We assessed social and community context through interpersonal network size, household members, marital status, and social activity frequency to reflect potential social isolation and civic participation [26,41]. We summed items within the social and community context and built environment domains, yielding a domain score ranging from 0 to 1 with equal weighting for each component; scores in the highest quintile indicate domain-level disadvantage [42,43].

The cumulative social disadvantage score was derived by summing these five continuous domain scores (each scaled from 0 to 1), yielding a total score ranging from 0 to 5 with equal weighting [34,42,44]. This score was divided into quintiles [42,45] (Table S2 in the **Online Supplementary Document**).

#### Healthcare and LTC service utilisation

Healthcare services included outpatient visits and hospital stays, while LTC services included formal and informal care. We quantified outpatient visits, formal care, and informal care as visit counts, while measuring hospital stays in days. We calculated healthcare services from clinical encounter records. We obtained LTC services from quarterly LTC insurance records.

#### Study covariates

Existing literature suggests that individual behavioural, social, and medical system determinants serve as the pivotal drivers facilitating the transition from general health status to CN status [7]. Covariates included age, gender, ethnicity, living arrangement, self-rated health status, functional impairment, disease influence on survivability, number of chronic conditions, visual impairment, and hearing impairment.

#### Statistical analysis

We summarised baseline characteristics across quintiles of the cumulative social disadvantage score. We presented continuous variables as means and standard deviations, and categorical variables as frequencies and percentages. We compared groups using the Pearson χ^2^ test for categorical variables and one-way analysis of variance for continuous variables.

Negative binomial regression models were chosen to account for data overdispersion, estimating care service utilisation across cumulative social disadvantage quintiles [46]. We conducted a linear trend test using the estimates to assess the relationship between increasing social disadvantage quintile and service utilisation. We constructed separate models to assess the association between each social disadvantage domain and care service utilisation. Multiplicative and additive interactions between the two domains with the highest incidence rate ratios (IRRs) identified in these models were evaluated by calculating the relative excess risk due to interaction (RERI), the attributable proportion due to interaction, and the synergy index (SI), along with their 95% confidence intervals (CIs) [47]. We also ran separate models for each social disadvantage item. All models were adjusted for potential confounding covariates. We assessed multicollinearity among these covariates using the variance inflation factor, with all values below the threshold of 5. Model fit, goodness-of-fit, and distributional adequacy were evaluated using the Akaike Information Criterion, Bayesian Information Criterion, pseudo-R^2^, root mean square error, and dispersion parameters [48]. We performed subgroup analyses to assess variation in this association across subpopulations.

We constructed parallel multiple mediation models with cumulative social disadvantage as the independent variable, formal and informal care as simultaneous mediators, and outpatient visit frequency and hospital stay duration as outcomes. A causal mediation analysis under a counterfactual framework was performed to decompose the total effect into the average causal mediation effect of individual and total mediators, and the average direct effect [49]. The proportion of the total effect mediated was quantified using the percentage mediated [50].

We conducted sensitivity analyses to assess the robustness of the findings. First, because previous studies have often operationalised cumulative social disadvantage using quartiles or the absolute count of disadvantages [45,51], we also applied these alternative approaches. Second, we annualised formal and informal care measures to align with the annual statistical period of healthcare services. Third, we excluded household members and marital status from the social context disadvantage to avoid potential multicollinearity and overlap with controlled variables such as living arrangements.

We used *R*, version 4.5.1 (R Core Team, Vienna, Austria) for all analyses, with a *P*-value <0.05 considered statistically significant.

## RESULTS

### Baseline characteristics

We included 42,499 participants, with a mean age of 80.72 years (standard deviation = 8.42). Among the dimensions of social disadvantage, built environment disadvantage accounted for the highest proportion (35.5%), followed by educational disadvantage (26.0%), while economic disadvantage accounted for the lowest proportion (5.0%) (**Table 1**).

**Table 1:** Baseline characteristics of the study population across social disadvantage quintiles*

	Overall (n = 42,499)	Quintile 1 (n = 8500)	Quintile 2 (n = 8500)	Quintile 3 (n = 8500)	Quintile 4 (n = 8500)	Quintile 5 (n = 8499)	*P*-value
**Age in years**							<0.001
60–74	10,539 (24.8)	2,607 (30.7)	2,852 (33.6)	2,051 (24.1)	1,502 (17.7)	1,527 (18.0)	
75–89	25,165 (59.2)	5,133 (60.4)	4,684 (55.1)	4,992 (58.7)	5,051 (59.4)	5,305 (62.4)	
≥90	6,795 (16.0)	760 (8.9)	964 (11.3)	1,457 (17.1)	1,947 (22.9)	1,667 (19.6)	
**Sex**							<0.001
Female	24,575 (57.8)	3,582 (42.1)	4,404 (51.8)	5,171 (60.8)	5,750 (67.6)	5,668 (66.7)	
Male	17,924 (42.2)	4,918 (57.9)	4,096 (48.2)	3,329 (39.2)	2,750 (32.4)	2,831 (33.3)	
**Ethnicity**							<0.001
Han Chinese	40,840 (96.1)	7,840 (92.2)	8,093 (95.2)	8,248 (97.0)	8,296 (97.6)	8,363 (98.4)	
Other ethnic minorities	1,659 (3.9)	660 (7.8)	407 (4.8)	252 (3.0)	204 (2.4)	136 (1.6)	
**Living arrangement**							<0.001
Home-based	38,110 (89.7)	7,591 (89.3)	7,622 (89.7)	7,375 (86.8)	7,622 (89.7)	7,900 (93.0)	
Healthcare institution	10 (0.0)	3 (0.0)	1 (0.0)	1 (0.0)	3 (0.0)	3 (0.0)	
Nursing home	4,379 (10.3)	906 (10.7)	877 (10.3)	1,124 (13.2)	876 (10.3)	596 (7.0)	
**Functional impairment**							<0.001
Mildly impairment	9,521 (22.4)	2,223 (26.2)	1,820 (21.4)	1,657 (19.5)	1,795 (21.1)	2,026 (23.8)	
Moderately impairment	14,741 (34.7)	3,237 (38.1)	3,146 (37.0)	2,978 (35.0)	2,733 (32.2)	2,647 (31.1)	
Severe impairment	18,237 (42.9)	3,040 (35.8)	3,534 (41.6)	3,865 (45.5)	3,972 (46.7)	3,826 (45.0)	
**Visual impairment**							<0.001
No impairment	40,178 (94.5)	8,174 (96.2)	8,075 (95.0)	7,997 (94.1)	7,923 (93.2)	8,009(94.2)	
Impairment	2,321 (5.5)	326 (3.8)	425 (5.0)	503 (5.9)	577 (6.8)	490 (5.8)	
**Hearing impairment**							<0.001
No impairment	37,382 (88.0)	7,831 (92.1)	7,628 (89.7)	7,338 (86.3)	7,085 (83.3)	7,503 (88.3)	
Impairment	5,117 (12.0)	669 (7.9)	872 (10.3)	1,162 (13.7)	1,418 (16.7)	996 (11.7)	
**Number of chronic diseases**							<0.001
3	25,465 (59.9)	4,912 (57.8)	4,856 (57.1)	4,996 (58.8)	5,205 (61.2)	5,496 (64.7)	
4	12,932 (30.4)	2,658 (31.3)	2,680 (31.5)	2,648 (31.2)	2,541 (29.9)	2,405 (28.3)	
5	3,357 (7.9)	767 (9.0)	784 (9.2)	700 (8.2)	608 (7.2)	498 (5.9)	
≥6	745 (1.8)	163 (1.9)	180 (2.1)	156 (1.8)	146 (1.7)	100 (1.2)	
**Self-rated health status, x̄ (SD)**	2.06 (0.61)	2.03 (0.56)	2.01 (0.57)	2.01 (0.59)	2.06 (0.63)	2.22 (0.68)	
**Disease influence on survivability**							0.028
No disease or negligible impact	532 (1.3)	110 (1.3)	93 (1.1)	103 (1.2)	126 (1.5)	100 (1.2)	
Moderate impact on daily functioning	17,506 (41.2)	3,586 (42.2)	3,531 (41.5)	3,535 (41.6)	3,450 (40.6)	3,404 (40.1)	
Severe symptoms, clinically stable	18,931 (44.5)	3,725 (43.8)	3,775 (44.4)	3,819 (44.9)	3,794 (44.6)	3,818 (44.9)	
Severe, unstable, life-threatening potential	5,341 (12.6)	1,051 (12.4)	1,061 (12.5)	999 (11.8)	1,097 (12.9)	1,133 (13.3)	
Critical, terminal stage	189 (0.4)	28 (0.3)	40 (0.5)	44 (0.5)	33 (0.4)	44 (0.5)	
**Cumulative social disadvantage, x̄ (SD)**	2.09 (0.75)	1.15 (0.22)	1.63 (0.10)	1.99 (0.11)	2.42 (0.15)	3.25 (0.37)	<0.001
**Economic disadvantage**							<0.001
Yes	2,109 (5.0)	1 (0.0)	4 (0.0)	13 (0.2)	102 (1.2)	1,989 (23.4)	
No	40,390 (95.0)	8,499 (100.0)	8,496 (100.0)	8,487 (99.8)	8,398 (98.8)	6,510 (76.6)	
**Educational disadvantage**							<0.001
Yes	11,029 (26.0)	8 (0.0)	149 (1.8)	1,275 (15.0)	3,236 (38.1)	6,361 (74.8)	
No	31,470 (74.0)	8,492 (99.9)	8,351 (98.2)	7,225 (85.0)	5,264 (61.9)	2,138 (25.2)	
**Healthcare access disadvantage**							<0.001
Yes	4,467 (10.5)	5 (0.0)	25 (0.3)	137 (1.6)	425 (5.0)	3,875 (45.6)	
No	38,032 (89.5)	8,495 (99.9)	8,475 (99.7)	8,363 (98.4)	8,075 (95.0)	4,624 (54.4)	
**Built environment disadvantage**							<0.001
Yes	15,069 (35.5)	657 (7.7)	1,575 (18.5)	2,745 (32.3)	4,308 (50.7)	5,784 (68.1)	
No	27,430 (64.5)	7,843 (92.3)	6,925 (81.5)	5,755 (67.7)	4,192 (49.3)	2,715 (31.9)	
**Social and community context disadvantage**							<0.001
Yes	9,492 (22.3)	377 (4.4)	1,113 (13.1)	1,986 (23.4)	2,908 (34.2)	3,108 (36.6)	
No	33,007 (77.7)	8,123 (95.6)	7,387 (86.9)	6,514 (76.6)	5,592 (65.8)	5,391 (63.4)	
**Frequency of outpatient visits, x̄ (SD)**	6.79 (10.11)	8.69 (10.54)	8.41 (10.75)	7.65 (10.83)	6.44 (10.09)	2.77 (6.50)	<0.001
**Duration of hospital stays, x̄ (SD)**	7.25 (14.58)	8.32 (15.72)	8.51 (15.87)	7.88 (15.15)	6.93 (14.01)	4.64 (11.34)	<0.001
**Frequency of formal care, x̄ (SD)**	11.30 (16.09)	13.15 (16.60)	12.49 (16.85)	12.48 (17.16)	11.11 (15.02)	7.29 (13.91)	<0.001
**Frequency of informal care, x̄ (SD)**	20.89 (24.40)	21.85 (25.12)	23.31 (26.05)	22.06 (24.31)	22.19 (24.07)	15.06 (21.32)	<0.001

SD – standard deviation, LTC – long-term care, x̄ – mean

*Values presented as absolute numbers (n) and weighted percentages (%) unless specified otherwise.

### Association between cumulative social disadvantage and care service utilisation

Greater cumulative social disadvantage, as represented by higher social disadvantage quintiles, was significantly associated with lower care service utilisation (*P* for trend*<*0.001) (**Figure 2**, Panels A and B). Specifically, compared with participants with the lowest social disadvantage (Quintile 1), those with the highest social disadvantage (Quintile 5) exhibited a 67.1% lower rate of outpatient visits (IRR = 0.329; 95% CI = 0.313–0.346), a 36.9% lower rate of hospital stays (IRR = 0.631; 95% CI = 0.580–0.687), a 46.7% lower rate of formal care utilisation (IRR = 0.533; 95% CI = 0.527–0.538), and a 32.9% lower rate of informal care utilisation (IRR = 0.671; 95% CI = 0.632–0.713) (Table S3 in the **Online Supplementary Document**).

**Figure 2:**
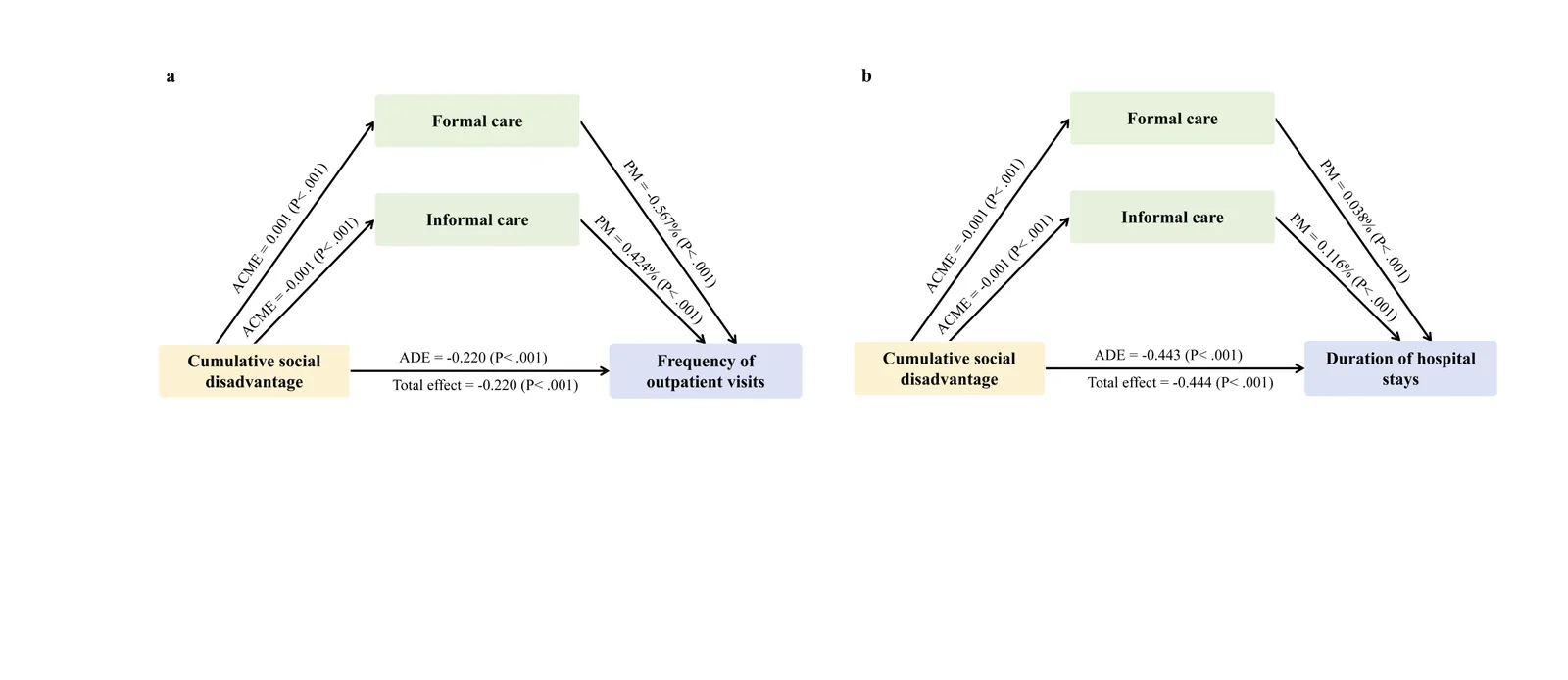
Associations between cumulative social disadvantage and service utilisation. **Panel A.** Cumulative social disadvantage and healthcare service utilisation. **Panel B.** Cumulative social disadvantage and LTC utilisation. ACME – average causal mediation effect, ADE – average direct effect, LTC – long-term care, PM – proportion mediated.

### Association between social disadvantage domains and care service utilisation

Economic stability, education, healthcare access, and social and community context were significantly associated with healthcare service utilisation in the preceding 12 months (**Table 2**). Compared with participants in Quintile 1, educational disadvantage (IRR = 0.668; 95% CI = 0.641–0.697) and healthcare access disadvantage (IRR = 0.463; 95% CI = 0.426–0.504) showed the strongest inverse associations with outpatient visit frequency. Similarly, educational (IRR = 0.781; 95% CI = 0.728–0.839) and healthcare access disadvantage (IRR = 0.591; 95% CI = 0.539–0.650) showed substantial negative associations with hospital stays.

**Table 2:** Associations between social disadvantage domains and care service utilisation*

	Frequency of outpatient visits		Duration of hospital stay		Frequency of formal care		Frequency of informal care	
	**(IRR, 95% CI)**	***P*-value**	**(IRR, 95% CI)**	***P*-value**	**(IRR, 95% CI)**	***P*-value**	**(IRR, 95% CI)**	***P*-value**
**Economic disadvantage**	0.755 (0.702–0.813)	<0.001	0.812 (0.775–0.972)	<0.001	1.675 (1.518–1.853)	<0.001	1.852 (1.704–2.016)	<0.001
**Educational disadvantage**	0.668 (0.641–0.697)	<0.001	0.781 (0.728–0.839)	<0.001	0.689 (0.650–0.731)	<0.001	0.868 (0.796–0.939)	<0.001
**Healthcare access disadvantage**	0.463 (0.426–0.504)	<0.001	0.591 (0.539–0.650)	<0.001	0.593 (0.530–0.666)	<0.001	0.497 (0.451–0.549)	<0.001
**Built environment disadvantage**	1.096 (0.996–1.209)	0.061	1.181 (1.003–1.402)	0.054	0.928 (0.814–1.064)	0.291	1.365 (1.221–1.531)	<0.001
**Social and community context disadvantage**	0.926 (0.891–0.963)	<0.001	0.924 (0.865–0.988)	<0.05	0.980 (0.930–1.033)	0.463	0.998 (0.954–1.045)	0.932
**AIC**	231,966.15		188,520.40		241,694.03		306,997.45	
**BIC**	232,191.24		188,745.53		241,919.12		307,222.54	
**R^2^**	0.211		0.164		0.168		0.130	
**RMSE**	9.708		10.248		22.916		14.637	
**Dispersion ratio**	1.066		0.795		0.92		1.064	

AIC – Akaike information criterion, BIC – Bayesian information criterion, CI – confidence interval, IRR – incidence rate ratio, R^2^ – pseudo-R^2^, RMSE – root mean square error

*The lowest cumulative social disadvantage group is the reference group. Adjusted for age, gender, ethnicity, living arrangement, self-rated health status, activities of daily living, number of chronic diseases, disease influence on survivability, visual impairment, and hearing impairment.

Economic stability, education, and healthcare access were significantly associated with LTC service utilisation in the preceding three months. Compared with Quintile 1, educational disadvantage (IRR = 0.689; 95% CI = 0.659–0.731) and healthcare access disadvantage (IRR = 0.593; 95% CI = 0.530–0.666) showed the strongest inverse associations with formal care frequency. Similarly, educational (IRR = 0.497; 95% CI = 0.451–0.549) and healthcare access disadvantage (IRR = 0.591; 95% CI = 0.539–0.650) showed substantial negative associations with informal care frequency. However, economic disadvantage was associated with an increased formal care frequency (IRR = 1.675; 95% CI = 1.518–1.853) and informal care frequency (IRR = 1.852; 95% CI = 1.704–2.016).

We also conducted regression analyses examining the associations between specific items within each domain and care service utilisation (Table S4 in the **Online Supplementary Document**).

### Interactive associations of educational and healthcare access disadvantages with care service utilisation

Educational and healthcare access disadvantages demonstrated significant multiplicative and additive interactions (**Table 3**). On the multiplicative scale, significant interactions were observed for the frequency of outpatient visits (IRR = 0.124; 95% CI = 0.110–0.140), duration of hospital stay (IRR = 0.216; 95% CI = 0.180–0.260), frequency of formal care (IRR = 0.223; 95% CI = 0.192–0.258), and frequency of informal care (IRR = 0.221; 95% CI = 0.195–0.251). Furthermore, significant positive additive interaction for service utilisation was observed for outpatient visits (RERI = 2.427; 95% CI = 1.133–3.721), hospital stays (RERI = 3.547; 95% CI = 2.539–4.555), formal care (RERI = 2.101; 95% CI = 1.202–3.000), and informal care (RERI = 3.480; 95% CI = 2.792–4.167). The attributable proportion due to interaction showed that the interaction accounted for 9.3% of the effect for outpatient visits, 56.7% for hospital stays, 19.1% for formal care, and 37.9% for informal care. Finally, SI significantly exceeded unity.

**Table 3:** Measures for estimation of the interaction between educational and healthcare access disadvantages for care service utilisation*

	Multiplicative interaction	Additive interaction		
	**IRR (95% CI)**	**RERI (95% CI)**	**AP (95% CI)**	**SI (95% CI)**
**Frequency of outpatient visits**	0.124 (0.110–0.140)	2.427 (1.133–3.721)	0.093 (0.044–0.142)	1.107 (1.045–1.170)
**Duration of hospital stay**	0.216 (0.180–0.260)	3.547 (2.539–4.555)	0.567 (0.418–0.717)	3.081 (1.387–4.775)
**Frequency of formal care**	0.223 (0.192–0.258)	2.101 (1.202–3.000)	0.191 (0.111–0.272)	1.267 (1.125–1.409)
**Frequency of informal care**	0.221 (0.195–0.251)	3.480 (2.792–4.167)	0.379 (0.308–0.449)	1.739 (1.499–1.979)

AP – attributable proportion, CI – confidence interval, IRR – incidence rate ratio, RERI – relative excess risk due to interaction, SI – synergy index

*All *P* < 0.001.

### Mediating effect of LTC services between cumulative social disadvantage and healthcare service utilisation

For outpatient visit frequency, the mediating proportions were −0.567% for formal and 0.424% for informal care. Similarly, for hospital stays, the mediating proportions were 0.038% for formal care and 0.116% for informal care (**Figure 3**, Panels A and B).

**Figure 3:**
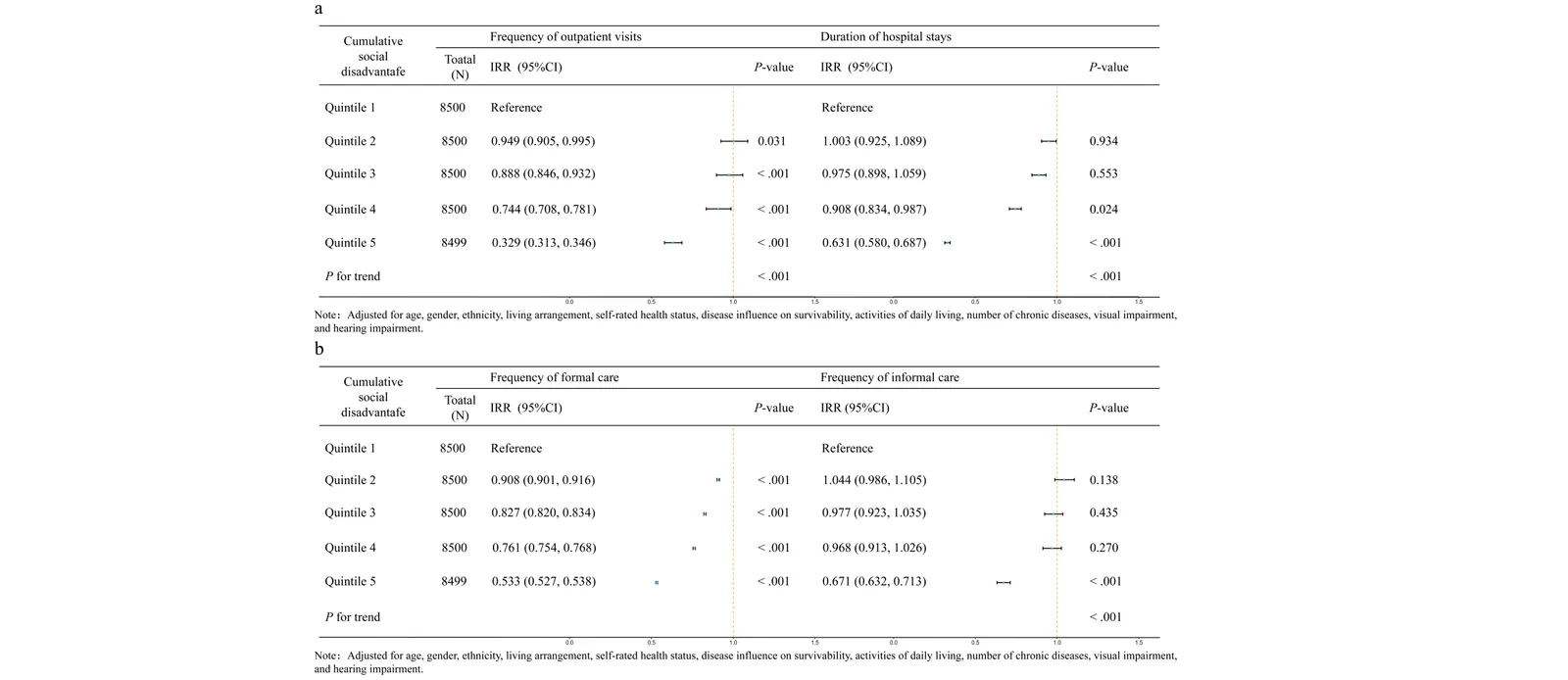
Mediating effects of long-term care services in the association between social disadvantage and healthcare service utilisation. **Panel A.** Mediating effects on outpatient visit utilisation. **Panel B.** Mediating effects on hospital stays. ACME – average causal mediation effect, ADE – average direct effect, PM – proportion mediated. Adjusted for age, gender, ethnicity, living arrangement, self-rated health status, activities of daily living, number of chronic diseases, disease influence on survivability, visual impairment, and hearing impairment.

### Sensitivity analysis

First, quartiles or absolute counts confirmed that higher cumulative social disadvantage significantly reduced utilisation of healthcare and LTC services. Second, after standardising LTC service utilisation into annual metrics, the conclusions remained unchanged. Finally, excluding marital status and household size from the social and community context domain did not substantially alter the results (Tables S5–9 in the **Online Supplementary Document**).

## DISCUSSION

In this cross-sectional study of 42,499 CN older adults, we investigated the association between social disadvantage and the utilisation of healthcare and LTC services. We found that increasing social disadvantage was progressively associated with lower utilisation of care services. Among the dimensions of social disadvantage, educational disadvantage and healthcare accessibility disadvantage had the strongest negative impacts, with a significant synergistic effect between them. Conversely, economic disadvantage was positively associated with hospital stays and LTC service utilisation. Furthermore, LTC services did not mediate the link between social disadvantage and healthcare utilisation.

Although prior studies highlight the widespread vulnerability of CN older adults, whose use of care services is easily compromised by socioeconomic factors [52], we found a weak negative association between low-to-moderate cumulative social disadvantage and care service use. This is likely because, for this population, high care needs are a critical survival priority that compels resource mobilisation, buffering against barriers typically imposed by social disadvantage [1]. However, as cumulative social disadvantage increases, its negative association with care service utilisation shows a clear dose-response relationship, consistent with previous findings [26,53]. This may be because severe social disadvantage entails profound multidimensional resource scarcity. Even as health needs continue to escalate, multiple practical barriers ultimately limit actual service utilisation [54–56]. Notably, our findings contrast with a USA study of CN populations, which found that social disadvantage was associated with a significant increase in care service utilisation [10]. In the USA study, participants were primarily members of integrated healthcare systems with comprehensive insurance coverage, where streamlined care coordination and financial protection allowed individuals to readily translate their health needs into increased healthcare utilisation [57,58]. Conversely, participants in our study lack access to a well-established healthcare and LTC system, which exacerbates the suppressive association between social disadvantage and care service utilisation [59].

We found that cumulative social disadvantage is most strongly associated with lower outpatient visit frequency. Given that older adults’ health management heavily relies on sustained and periodic outpatient visits, cumulative social disadvantage may disrupt this crucial continuity of care [1,2]. Notably, the negative association between cumulative social disadvantage and hospitalisation differs from that observed for outpatient visits. Hospital stays are primarily driven by involuntary and inelastic medical needs, typically triggered by health shocks [60]. In contrast, outpatient visits mainly support routine chronic disease management, where individuals have greater autonomy over their treatment-seeking decisions. Consequently, socially disadvantaged older adults are more likely to forgo these visits, driving the pronounced negative association.

Among dimensions of social disadvantage, educational disadvantage and healthcare access disadvantage exhibited the most pronounced negative associations with care service utilisation among CN older adults, consistent with previous findings [18,61]. Individuals with educational disadvantages not only struggle to translate latent health needs into actual healthcare utilisation [62], but also encounter hidden barriers when navigating complex LTC application procedures, eligibility assessments, and resource coordination [25]. Furthermore, individuals with healthcare access disadvantage face high costs to overcome spatial distances due to a lack of nearby primary healthcare facilities, compounded by physical functional limitations. Moreover, the lack of necessary companion support further hinders CN older adults from obtaining essential care services [63]. The intersection of these two disadvantages creates a synergistic effect, further compounding barriers to utilising care services. Therefore, targeted interventions should prioritise CN older adults experiencing both disadvantages.

Interestingly, economic disadvantage was associated with a greater utilisation of LTC services. The LTC insurance in China primarily targets insured individuals whose daily activities are limited by ageing or illness, providing financial protection for home-based or institutional care [64]. This initiative lowers the financial barriers for economically disadvantaged populations to access LTC services. For economically disadvantaged CN older adults, who often have greater care needs, LTC insurance may enable access to more care services. Furthermore, built environment disadvantage was positively associated with the utilisation of informal care services. This may be because built environment disadvantages create physical barriers to older adults’ daily self-care and outdoor activities, which may coincide with diminished capacity for independent living [65]. Those facing such environmental constraints may rely more heavily on home-based care services provided through the LTC insurance system to fulfil daily care needs.

While prior literature indicates that LTC services can substitute for healthcare service utilisation [66], we found that LTC services have a negligible indirect association with healthcare service utilisation among CN older adults. For CN older adults who face high healthcare expenditures and intensive demands for LTC, both services represent rigid demands [2]. The primary focus of LTC services is daily living assistance, whereas medical services concentrate on disease diagnosis and clinical treatment, representing distinct service domains [67]. Therefore, strategies addressing health disparities related to social disadvantage may need to target both sectors independently.

Sensitivity analyses showed a gradient between social disadvantage and the utilisation of healthcare and LTC services, with consistent trends observed regardless of whether social disadvantage was measured by quintiles, quartiles, or absolute counts. Excluding marital status and household size from the social and community context domain did not substantially alter the core findings. Standardising LTC service utilisation metrics to an annual basis yielded robust results, ensuring comparability between healthcare and LTC service utilisation. These findings confirm that the observed results are not driven by methodological artefacts, such as categorisation criteria, indicator selection, or temporal measurement discrepancies.

### Practical implications of the findings

Our results offer several practical implications, but they should be interpreted with caution given the observational, cross-sectional design. First, healthcare services should establish a standardised screening mechanism for social disadvantage to early identify social disadvantages among CN older adults, with a particular focus on educational disadvantages and barriers to healthcare access [68]. Second, given the significant negative association between social disadvantages and outpatient visit utilisation, and considering that outpatient visit utilisation is closely related to disease progression, greater attention should be paid to this issue [69]. Finally, given the intensive demands for both healthcare and LTC services, alongside the weak mediating effect, the provision of these services should be synergistically advanced to achieve mutual system reinforcement [2].

### Limitations

Several limitations should be considered. First, the cross-sectional design precludes causal inference between social disadvantage and care service utilisation, and we cannot rule out potential reverse causality. Second, while CN status based on established criteria effectively identifies the target population, this may still overlook populations with fewer chronic conditions or normal functional status who nevertheless experience high care needs. Third, we derived the data from Shanghai, China, which limits the generalisability of the findings to other regions or to populations outside of LTC service applicants. Additionally, although we adjusted for multiple covariates, including cognitive impairment, household income, and insurance type, we cannot rule out residual confounding. Furthermore, not controlling for certain variables, including cognitive impairment, household income, and insurance type, may limit the interpretation of our findings. Excluding neighbourhood environments, caregiving resources, and regional disparities also limits our assessment of broader social disadvantage. Moreover, we measured healthcare services annually, whereas we measured LTC services quarterly, which may bias comparisons of utilisation. Finally, while sensitivity analyses tested different categorisations of cumulative social disadvantage, the equal-weighting approach failed to account for the relative importance of different dimensions.

## CONCLUSIONS

We provide empirical evidence of negative associations between cumulative social disadvantage and utilisation of healthcare and LTC services, and reveal a limited indirect association involving LTC services. Among the dimensions of social disadvantage, educational disadvantage and healthcare accessibility disadvantage had the strongest negative effects on service utilisation, with a synergistic effect between them. As a cross-sectional study, these findings cannot establish causal effects and should be examined in future longitudinal research. Nevertheless, by highlighting the importance of addressing social disadvantage, they can help inform targeted efforts to optimise care allocation for CN older adults.

## Additional material


Online Supplementary Document

## Data Availability

**Data availability:** Since the data used in this study were derived from the administrative Long-Term Care Insurance database managed by the Shanghai Healthcare Security Bureau, they are not publicly available due to data privacy laws and administrative regulations.
