# Supplementary material for: Cumulative social disadvantage and inequitable utilisation of healthcare and long-term care services among older adults with complex needs: a cross-sectional study in China
Source: J Glob Health. 2026 Oct 30;16:04352. doi: 10.7189/jogh.16.04352 (PMC13721841; doi:10.7189/jogh.16.04352)
Supplement: Supplementary file 1 [file jogh-16-04352-s001.pdf]

Supplement to: Zhang P, Xu J, Xu J, Zhu B, Ding H, Zhou L, Zhang Y. Cumulative social disadvantage and inequitable utilisation of healthcare and long-term care services among older adults with complex needs: a cross-sectional study in China. J Glob Health. 2026;16:04352.

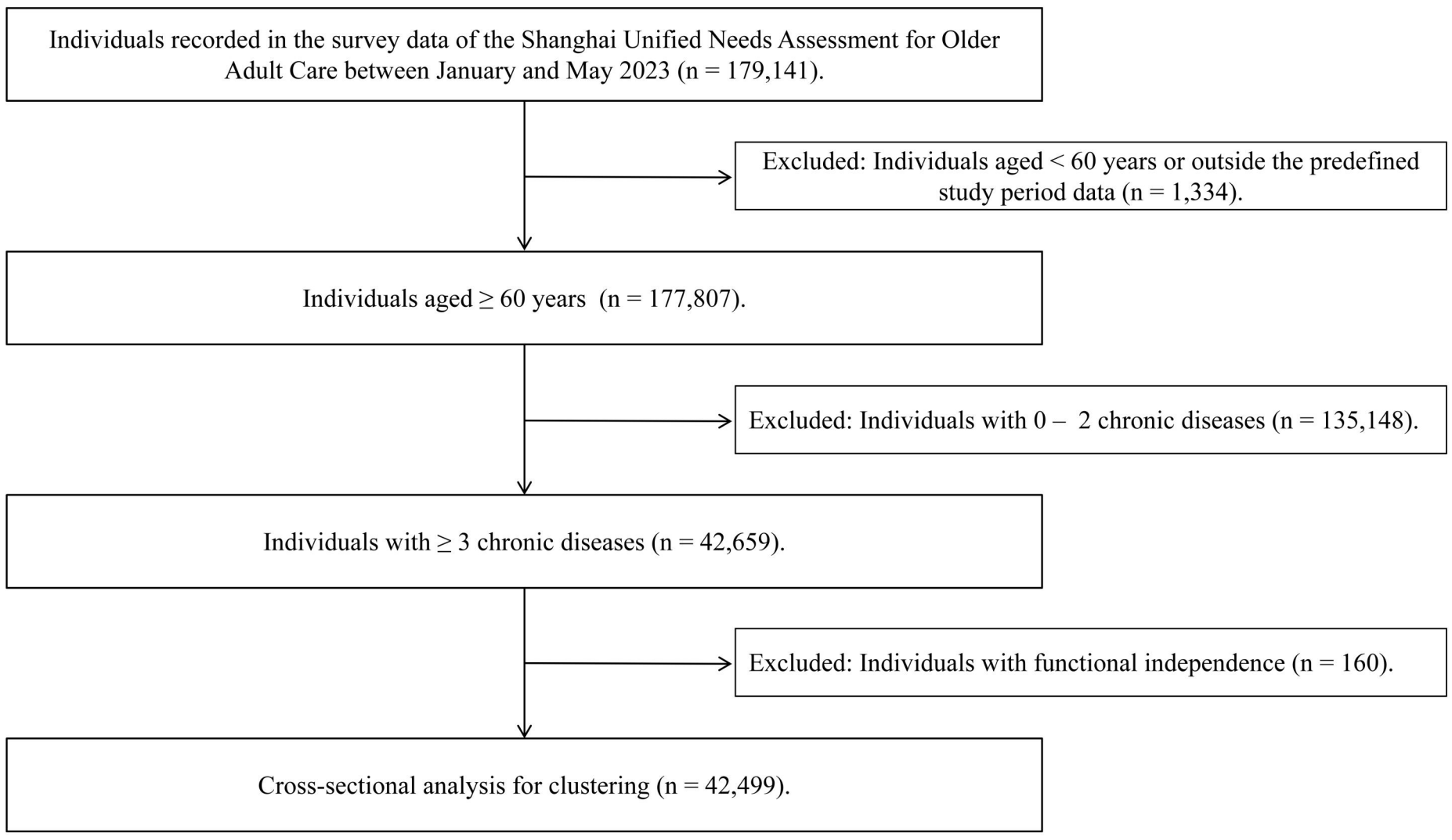

Figure S1. Participant flow diagram.

Table S1. Definitions of chronic disease categories for patients with complex needs.

Chronic Conditions	Source 1[1]	Source 2[2]	Inclusion
Acute myocardial infarction	√		
Ischemic heart disease	√	√	√
Chronic kidney disease	√	√	√
Congestive heart failure	√	√	√
Dementia	√	√	√
Diabetes	√	√	√
Chronic lung disease	√	√	√
Psychiatric disease	√	√	√
Specified heart arrhythmias	√	√	√
Cerebrovascular Disease	√	√	√
Amputation status	√		
Arthritis and other inflammatory tissue diseases	√	√	√
Artificial openings	√		
Benign prostatic hyperplasia	√		
Cancer	√	√	√
Cystic fibrosis	√		
Endocrine and metabolic disorders	√	√	√
Eye disease	√		
Hematological disease	√	√	√
Hyperlipidemia	√		
Hypertension	√	√	√
Immune disorders	√	√	√
Inflammatory bowel disease	√		
Liver and biliary disease	√	√	√
Neuromuscular disease	√	√	√
Osteoporosis	√		
Paralytic diseases/conditions	√	√	√
Skin ulcer	√		
Substance abuse	√	√	√
Connective tissue disorders		√	
Thyroid disease	√		
Severe Mental Illness		√	
HIV / AIDS		√	

Table S2. Social disadvantages based Department of Health and Human Services and the Healthy People 2030 framework.

**Domain**	**Survey domain** [a]	**Measures used**	**Score**	**Social disadvantage**
Economic stability	Economic source	Stable pension income	Unavailable = 1, Available = 0	Absence of a stable pension income [b]
Education access and quality	Education level	Highest educational attainment	Illiterate = 1, Primary school = 0.75, Junior high school = 0.50, Senior high school = 0.25, University or above = 0	Illiteracy [c]
Healthcare access	Access to primary care	Community health centers within 1 km	Unavailable = 0.50, Available = 0	Unavailability of both
	Access to healthcare services	Outpatient visit accompaniment	Unavailable = 0.50, Available = 0	
Built environment	Quality of housing	Indoor steps	Present = 0.20, Absent = 0	Cumulative built environment disadvantage (score ≥ 0.80) [d]
		Elevator in the building	Unavailable = 0.20, Available = 0	
		Grab bars	Unavailable = 0.20, Available = 0	
		Bathroom grab bars	Unavailable = 0.20, Available =0	
		Household cleanliness and tidiness	Poorly maintained = 0.20, Well-maintained = 0	
Social and community context	Social isolation and civic participation	Marital status	Without spouse = 0.25, With spouse=0	Cumulative built environment disadvantage (score ≥ 0.80) [e]
		Number of household members	0 person = 0.25, 1 person = 0.125, ≥ 2 persons = 0	
		Interpersonal network size	0–3 persons = 0.25, 4–9 persons = 0.125, ≥ 10 persons = 0	
		Frequency of social activities	Multiple times daily or once daily = 0.25, Once weekly = 0.125, Once monthly or less than once monthly = 0	
Cumulative Social disadvantage score	All of the above	All of the above	A cumulative score was obtained by summing the number of points (range 0–5).	-

[a] Indicate Unified Needs Assessment Form for Older Adults Care Survey Survey.

[b] Detailed individual income data were omitted due to privacy sensitivities, and economic stability was evaluated based on pension status.

[c] Based on national data reporting a 67.0% illiteracy rate among Chinese older adults aged 80 and over, illiteracy was designated as a key social disadvantage factor.

[d] Built environment score ranged from 0 to 0.20, with a cumulative score of ≥0.80 (indicating at least four adverse conditions) defined as cumulative built environment disadvantage.

[e] Social and community context score ranged from 0 to 0.25, with a cumulative score of ≥0.80 (indicating at least four adverse conditions) defined as cumulative built environment disadvantage.

Table S3. Subgroup analysis of the associations between social disadvantage and service utilization.

Measures	Frequency of outpatient visits (IRR, 95% CI)	Duration of hospital stay (IRR, 95% CI)	Frequency of formal care (IRR, 95% CI)	Frequency of informal care (IRR, 95% CI)
Age (Ref: 60-74)				
P for interaction	0.027	<0.001	<0.001	<0.001
75-89	0.897***	0.776***	1.269***	1.027*
	(0.869, 0.926)	(0.744, 0.810)	(1.226, 1.314)	(1.000, 1.055)
≥ 90	0.736***	0.626***	1.754***	1.012
	(0.702, 0.772)	(0.587, 0.668)	(1.681, 1.831)	(0.976, 1.049)
Sex (Ref: Male)				
P for interaction	0.722	0.003	<0.001	<0.001
Female	1.073***	0.750***	1.185***	1.009
	(1.043, 1.104)	(0.722, 0.780)	(1.152, 1.218)	(0.987, 1.032)
Ethnicity (Ref: Han Chinese)				
P for interaction	0.073	0.591	0.004	0.234
Other ethnic minorities	1.092**	0.889*	0.910**	0.542***
	(1.024, 1.165)	(0.805, 0.981)	(0.848, 0.976)	(0.504, 0.584)
Living arrangement (Ref: Home-based)				
P for interaction	0.081	0.004	<0.001	<0.001
Healthcare institution	0.616	2.003	1.317	0.668
	(0.205, 1.847)	(0.874, 4.591)	(0.606, 2.860)	(0.290, 1.539)
Nursing home	0.491***	0.765***	3.301***	0.321***
	(0.463, 0.521)	(0.715, 0.819)	(3.211, 3.393)	(0.303, 0.340)
Number of chronic diseases (Ref: 3)				
P for interaction	<0.001	0.439	0.268	0.002
4	1.054***	1.101***	1.043**	0.944***
	(1.022, 1.087)	(1.056, 1.148)	(1.013, 1.075)	(0.921, 0.967)
5	1.117***	1.210***	1.044	0.879***
	(1.064, 1.174)	(1.133, 1.292)	(0.993, 1.097)	(0.842, 0.918)
≥ 6	1.178***	1.127	1.001	0.797***
	(1.071, 1.296)	(0.985, 1.289)	(0.904, 1.108)	(0.728, 0.873)
Functional impairment (Ref: Mild impairment)				
P for interaction	<0.001	0.007	<0.001	<0.001
Moderate impairment	0.936***	1.173***	1.579***	1.157***
	(0.902, 0.972)	(1.108, 1.242)	(1.508, 1.653)	(1.122, 1.194)
Severe impairment	0.818***	1.484***	2.973***	1.049**
	(0.787, 0.851)	(1.403, 1.570)	(2.846, 3.106)	(1.016, 1.082)
Visual impairment (Ref: No impairment)				
P for interaction	0.029	0.182	<0.001	<0.001
Impairment	1.037	0.827***	1.129***	1.033
	(0.977, 1.101)	(0.757, 0.903)	(1.068, 1.194)	(0.985, 1.083)

Hearing impairment (Ref: No impairment)				
P for interaction	<0.001	0.016	<0.001	<0.001
Impairment	0.893*** (0.854, 0.934)	0.916** (0.863, 0.972)	1.265*** (1.217, 1.314)	0.995 (0.962, 1.029)
Disease influence on survivability (Ref: No disease or negligible impact)				
P for interaction	0.536	0.303	0.023	0.622
Moderate impact on daily functioning	1.063 (0.935, 1.209)	1.076 (0.902, 1.285)	0.962 (0.855, 1.083)	1.064 (0.961, 1.179)
Severe symptoms, clinically stable	1.052 (0.925, 1.195)	1.107 (0.927, 1.322)	0.925 (0.822, 1.041)	1.079 (0.974, 1.196)
Severe, unstable, life-threatening potential	1.084 (0.950, 1.238)	1.079 (0.899, 1.295)	0.841** (0.744, 0.951)	1.029 (0.926, 1.144)
Critical, terminal stage	1.106 (0.869, 1.408)	1.158 (0.836, 1.605)	0.931 (0.735, 1.178)	1.016 (0.834, 1.238)

Note：*$P < 0.05$; **$P < 0.01$; ***$P < 0.001$.

Table S4. Associations between sub-items of social disadvantage and service utilization.

Measures	Frequency of outpatient visits (IRR, 95% CI)	Duration of hospital stay (IRR, 95% CI)	Frequency of formal care (IRR, 95% CI)	Frequency of informal care (IRR, 95% CI)
**Economic disadvantage**				
Highest education (Illiterate)	0.488***	0.865*	1.028***	1.480***
	(0.456, 0.522)	(0.758, 0.987)	(1.021, 1.035)	(1.472, 1.487)
**Educational disadvantage**				
Stable pension income (Unavailable)	0.406***	0.591***	0.602***	0.757***
	(0.387, 0.427)	(0.539, 0.648)	(0.599, 0.605)	(0.754, 0.760)
**Healthcare access disadvantage**				
Community health centers within 1 km (Unavailable)	0.654***	0.757***	0.548***	0.575***
	(0.622, 0.687)	(0.697, 0.822)	(0.546, 0.551)	(0.573, 0.577)
Outpatient visit accompaniment (Unavailable)	0.287***	0.711***	0.554***	0.481***
	(0.268, 0.307)	(0.648, 0.779)	(0.551, 0.556)	(0.480, 0.483)
**Built environment disadvantage**				
Indoor steps	0.781***	0.980	0.784***	1.071***
(Represent)	(0.737, 0.827)	(0.877, 1.096)	(0.779, 0.789)	(1.065, 1.076)
Elevator in the building	0.728***	0.878***	0.727***	0.930***
(Unavailable)	(0.699, 0.758)	(0.820, 0.941)	(0.724, 0.729)	(0.927, 0.932)
Grab bars	0.754***	0.895**	0.738***	0.916***
(Unavailable)	(0.725, 0.785)	(0.838, 0.957)	(0.735, 0.741)	(0.914, 0.919)
Bathroom grab bars	0.738***	0.873***	0.692***	0.876***
(Unavailable)	(0.709, 0.769)	(0.816, 0.935)	(0.690, 0.695)	(0.874, 0.878)
Household cleanliness and tidiness	0.716***	0.850***	0.757***	0.910***
(Poorly maintained)	(0.686, 0.748)	(0.791, 0.912)	(0.754, 0.760)	(0.908, 0.913)
**Social context disadvantage**				
Interpersonal network size	0.744***	0.892**	0.832***	1.019***
(0-3 persons)	(0.709, 0.780)	(0.826, 0.963)	(0.828, 0.835)	(1.016, 1.022)
Number of household members	0.666***	0.766***	0.775***	0.785***
(0)	(0.636, 0.696)	(0.708, 0.830)	(0.772, 0.778)	(0.782, 0.787)
Marital status	0.702***	0.905**	0.775***	0.917***
(Without spouse)	(0.674, 0.731)	(0.846, 0.968)	(0.773, 0.778)	(0.915, 0.919)
Frequency of social activities	0.893***	0.859**	0.806***	0.737***
(Multiple times daily or once daily)	(0.846, 0.943)	(0.772, 0.956)	(0.800, 0.811)	(0.733, 0.740)

Note: The lowest cumulative social disadvantage group as reference. Adjusted for age, gender, ethnicity, living arrangement, self-rated health status, activities of daily living, burden of chronic diseases, visual impairment, and hearing impairment. *$P < 0.05$; **$P < 0.01$; ***$P < 0.001$.

Table S5. Sensitivity Analysis 1: Associations between cumulative social disadvantage quartiles and care service utilization *

Measures	Frequency of outpatient visits	Duration of hospital stay	Frequency of formal care	Frequency of informal care
	**(IRR, 95% CI)**	**(IRR, 95% CI)**	**(IRR, 95% CI)**	**(IRR, 95% CI)**
Quintile 1	Reference	Reference	Reference	Reference
Quintile 2	0.951** (0.911, 0.993)	0.999 (0.929, 1.075)	0.886*** (0.880, 0.893)	1.009 (0.959, 1.062)
Quintile 3	0.829*** (0.793, 0.866)	0.907** (0.842, 0.977)	0.801*** (0.794, 0.807)	0.956 (0.908, 1.008)
Quintile 4	0.403*** (0.385, 0.422)	0.691*** (0.640, 0.746)	0.585*** (0.580, 0.591)	0.724*** (0.685, 0.764)
P for trend	<0.001	<0.001	<0.001	<0.001

*Converting the cumulative social disadvantage quintiles into quartiles.

Table S6. Sensitivity Analysis 2: Associations between the number of cumulative social disadvantages and care service utilization *

Measures	Frequency of outpatient visits (IRR, 95% CI)	Duration of hospital stay (IRR, 95% CI)	Frequency of formal care (IRR, 95% CI)	Frequency of informal care (IRR, 95% CI)
0	Reference	Reference	Reference	Reference
1	0.708***<br>(0.683, 0.734)	0.913**<br>(0.858, 0.972)	0.839***<br>(0.833, 0.844)	0.957*<br>(0.916, 1.000)
2	0.381***<br>(0.363, 0.400)	0.560**<br>(0.516, 0.608)	0.659***<br>(0.652, 0.665)	0.755***<br>(0.712, 0.800)
3	0.177***<br>(0.162, 0.194)	0.369**<br>(0.321, 0.426)	0.534***<br>(0.525, 0.544)	0.667***<br>(0.605, 0.738)
4	0.425**<br>(0.246, 0.805)	0.524<br>(0.222, 1.692)	0.875***<br>(0.787, 0.970)	1.008<br>(0.544, 2.177)
P for trend	<0.001	<0.001	<0.001	<0.001

*Replacing the quintile-based classification of cumulative social disadvantage with the number of social disadvantaged domains.

Table S7. Sensitivity Analysis 3: Associations between cumulative social disadvantage and care service utilization *

**Measures**	**Frequency of outpatient visits**	**Duration of hospital stay**	**Frequency of formal care**	**Frequency of informal care**
	**(IRR, 95% CI)**	**(IRR, 95% CI)**	**(IRR, 95% CI)**	**(IRR, 95% CI)**
Quintile 1	Reference	Reference	Reference	Reference
Quintile 2	0.949* (0.905, 0.995)	1.003 (0.925, 1.089)	0.908*** (0.904, 0.912)	1.035*** (1.031, 1.038)
Quintile 3	0.888*** (0.846, 0.932)	0.975 (0.898, 1.059)	0.827*** (0.824, 0.831)	0.978*** (0.974, 0.981)
Quintile 4	0.744*** (0.708, 0.781)	0.908*** (0.835, 0.987)	0.761*** (0.757, 0.764)	0.953*** (0.949, 0.956)
Quintile 5	0.329*** (0.313, 0.346)	0.631*** (0.580, 0.687)	0.533*** (0.530, 0.535)	0.639*** (0.637, 0.641)
P for trend	<0.001	<0.001	<0.001	<0.001

* Converting the frequency outcome measures for formal care and informal care from quarterly to annual metrics.

Table S8. Sensitivity Analysis 4: Associations between cumulative social disadvantage and care service utilization *

Measures	Frequency of outpatient visits (IRR, 95% CI)	Duration of hospital stay (IRR, 95% CI)	Frequency of formal care (IRR, 95% CI)	Frequency of informal care (IRR, 95% CI)
Quintile 1	Reference	Reference	Reference	Reference
Quintile 2	0.916***<br>(0.874, 0.961)	1.050<br>(0.967, 1.140)	1.015***<br>(1.006, 1.024)	1.124***<br>(1.061, 1.189)
Quintile 3	0.840***<br>(0.800, 0.881)	0.989<br>(0.911, 1.074)	0.888***<br>(0.881, 0.896)	1.122***<br>(1.060, 1.188)
Quintile 4	0.744***<br>(0.708, 0.781)	0.950<br>(0.874, 1.033)	0.768***<br>(0.761, 0.776)	1.062*<br>(1.002, 1.126)
Quintile 5	0.317***<br>(0.302, 0.334)	0.675***<br>(0.620, 0.734)	0.627***<br>(0.620, 0.633)	0.761***<br>(0.717, 0.808)
P for trend	<0.001	<0.001	<0.001	<0.001

* Excluding marital status and number of household members from the social and community context domain.

Table S9. Sensitivity Analysis 4: Associations between social disadvantage domains and care service utilization *

Measures	Frequency of outpatient visits (IRR, 95% CI)	Duration of hospital stay (IRR, 95% CI)	Frequency of formal care (IRR, 95% CI)	Frequency of informal care (IRR, 95% CI)
Economic disadvantage	0.756*** (0.703, 0.813)	0.823*** (0.784, 0.985)	1.692*** (1.532, 1.871)	1.868*** (1.719, 2.033)
Educational disadvantage	0.668*** (0.641, 0.697)	0.781*** (0.727, 0.839)	0.686*** (0.647, 0.727)	0.862*** (0.791, 0.934)
Healthcare access disadvantage	0.464*** (0.427, 0.504)	0.585*** (0.532, 0.643)	0.589*** (0.527, 0.661)	0.494*** (0.449, 0.545)
Built environment disadvantage	1.097 (0.997, 1.210)	1.186* (1.007, 1.408)	0.931 (0.816, 1.067)	1.380*** (1.235, 1.548)
Social and community context disadvantage	0.889*** (0.860, 0.920)	1.041 (0.982, 1.103)	0.914 (0.864, 0.966)	1.003 (0.958, 1.051)

* Excluding marital status and number of household members from the social and community context domain

References

1. Long P, Abrams M, Milstein A, et al. Effective care for high-need patients. The Commonwealth Fund. WASHINGTON, DC: "National Academy of Medicine", 2021.
2. Ouayogodé MH, Mainor AJ, Meara E, et al. Association Between Care Management and Outcomes Among Patients With Complex Needs in Medicare Accountable Care Organizations. *JAMA Netw Open.* 2019; 2(7):e196939.

The STROBE reporting checklist

For checking that observational epidemiology research articles can be understood and used by everyone

How to use this reporting checklist

This reporting checklist allows authors to demonstrate that their manuscripts adhere to the STROBE reporting guideline.

If you have not used a reporting guideline before, read about how and why to use them and check whether STROBE is the most applicable reporting guideline for your work.

Reporting guidelines are most useful when used early in research. When writing a manuscript or application, consider using the full guidance where you'll find explanations and examples for each item.

After writing, demonstrate adherence by completing this checklist:

1. Specify where each item is described (see Note 1).
2. Cite this checklist (See Note 2).
3. Include your completed checklist as a supplement when submitting to a journal so that future readers can use it to find information.

	Item Description	Location (or reason for not reporting)
**Title and abstract**		
1a. Indicate the study's design	Indicate the study's design with a commonly used term in the title or the abstract.	Page 1, Line 1-3; and Page 2-3, Line 17-49
1b. Abstract	Provide in the abstract an informative and balanced summary of what was done and what was found.	Page 2-3, Line 17-49
**Introduction**		
2. Background / rationale	Explain the scientific background and rationale for the investigation being reported.	Page 4-5, Line 53-98
3. Objectives	State specific objectives, including any prespecified hypotheses.	Page 5, Line 99-109
**Methods**		
4. Study design	Present key elements of study design early in the paper.	Page 6, Line 112-123
5. Setting	Describe the setting, locations, and relevant dates, including periods of recruitment, exposure, follow-up, and data collection.	Page 6, Line 112-123
6a. Eligibility criteria	**Cohort study:** Give the eligibility criteria, and the	Page 6-7, Line 135-139

	sources and methods of selection of participants. Describe methods of follow-up. **Case-control study:** Give the eligibility criteria, and the sources and methods of case ascertainment and control selection. Give the rationale for the choice of cases and controls. **Cross-sectional study:** Give the eligibility criteria, and the sources and methods of selection of participants.	
6b. Matching criteria	**Cohort study:** For matched studies, give matching criteria and number of exposed and unexposed. **Case-control study:** For matched studies, give matching criteria and the number of controls per case.	NA
7. Variables	Clearly define all outcomes, exposures, predictors, potential confounders, and effect modifiers. Give diagnostic criteria, if applicable.	Page 7-8, Line 141-182
8. Data sources / measurement	For each variable of interest give sources of data and details of methods of assessment (measurement). Describe comparability of assessment methods if there is more than one group.	Page 7-8, Line 141-182
9. Bias	Describe any efforts to address potential sources of bias.	Page 9-10, Line 217-225
10. Study size	Explain how the study size was arrived at.	NA
11. Quantitative variables	Explain how quantitative variables were handled in the analyses. If applicable, describe which groupings were chosen, and why.	Page 8-9, Line 184-216
12a. Statistical methods	Describe all statistical methods, including those used to control for confounding.	Page 8-9, Line 184-228
12b. Statistical methods – subgroups and interactions	Describe any methods used to examine subgroups and interactions.	NA
12c. Statistical methods – missing data	Explain how missing data were addressed.	Page 6, Line 120-122
12di. Statistical methods – loss to follow-up	**Cohort study:** If applicable, describe how loss to follow-up was addressed.	NA
12dii. Statistical methods – matching cases and controls	**Case-control study:** If applicable, explain how matching of cases and controls was addressed.	NA
12diii. Statistical methods – sampling strategy	**Cross-sectional study:** If applicable, describe analytical methods taking account of sampling strategy.	Page 6, Line 120-122
12e. Statistical	Describe any sensitivity analyses.	Page 9-10, Line 217-225

methods – sensitivity analyses		
**Results**		
13a. Participant numbers	Report the numbers of individuals at each stage of the study—e.g., numbers potentially eligible, examined for eligibility, confirmed eligible, included in the study, completing follow-up, and analysed; Consider use of a flow diagram.	Page 10, Line 231-235
13b. Participants – non-participation	Give reasons for non-participation at each stage.	NA
13c. Participants – flow diagram	Consider use of a flow diagram.	Figure S1 in the Online Supplementary Document
14a. Descriptive data – participant characteristics	Give characteristics of study participants (e.g., demographic, clinical, social) and information on exposures and potential confounders. Present the information in a table.	Page 26-29, Table 1
14b. Descriptive data – missing data	Indicate the number of participants with missing data for each variable of interest.	NA
14c. Descriptive data – follow-up time	**Cohort study:** Summarise follow-up time—e.g., average and total amount.	NA
15. Outcome data	**Cohort study:** Report numbers of outcome events or summary measures over time. **Case-control study:** Report numbers in each exposure category, or summary measures of exposure. **Cross-sectional study:** Report numbers of outcome events or summary measures.	Page 10-12, Line 236-297
16a. Main results	Give unadjusted estimates and, if applicable, confounder-adjusted estimates and their precision (e.g., 95% confidence intervals). Make clear which confounders were adjusted for and why they were included.	Page 10-12, Line 236-297
16b. Main results – category boundaries	Report category boundaries when continuous variables were categorised.	Page 10-12, Line 236-297
16c. Main results – risk	If relevant, consider translating estimates of relative risk into absolute risk for a meaningful time period.	NA
17. Other analyses	Report other analyses done—e.g., analyses of subgroups and interactions, and sensitivity analyses.	Page 12, Line 299-306
**Discussion**		
18. Key results	Summarise key results with reference to study objectives.	Page 13, Line 308-318
19. Limitations	Discuss limitations of the study, taking into account sources of potential bias or imprecision. Discuss both	Page 16-17, Line 417-436

	direction and magnitude of any potential bias.	
20. Interpretation	Give a cautious overall interpretation considering objectives, limitations, multiplicity of analyses, results from similar studies, and other relevant evidence.	Page 13-16, Line 308-402
21. Generalisability	Discuss the generalisability (external validity) of the study results.	Page 16, Line 404-415
**Other information**		
22. Funding	Give the source of funding and the role of the funders for the present study and, if applicable, for the original study on which the present article is based.	Page 18, Line 460-464

1 How to specify where content is

Tell the reader where they can find information. E.g.,

- Results; paragraph 2
- Methods, Participants; paragraphs 1 & 2.
- Table 3
- Supplement B, para. 4

If you have chosen not to describe an item, explain why. You can do this in the checklist, or as a note below it.

You can describe items in the article body, or in tables, figures, or supplementary materials, and should prioritize items you feel are most important to your intended audience. The order of items in your manuscript does not need to match the order of items in this checklist. You can decide how best to structure your work.

2 How to cite

Describe how you used STROBE at the end of your Methods section, referencing the resources you used e.g.,

> 'We used the STROBE reporting guideline(1) to draft this manuscript, and the STROBE reporting checklist(2) when editing, included in supplement A'

If you use a reporting checklist, remember to include it as a supplement when publishing so that readers can easily find information and see how you have interpreted the guidance.

1. Elm E von, Altman DG, Egger M, Pocock SJ, Gøtzsche PC, Vandenbroucke JP, et al. The strengthening the reporting of observational studies in epidemiology (STROBE) statement: Guidelines for reporting observational studies. Annals of Internal Medicine [Internet]. 2007 Oct;147(8):573–7. Available from: https://www.acpjournals.org/doi/10.7326/0003-4819-147-8-200710160-00010

2. Elm E von, Altman DG, Egger M, Pocock SJ, Gøtzsche PC, Vandenbroucke JP, et al. The STROBE reporting checklist. In: Harwood J, Albury C, Beyer J de, Schlüssel M, Collins G, editors. The EQUATOR network reporting guideline platform [Internet]. The UK EQUATOR Centre; 2025. Available from: https:/resources.equator-network.org/reporting-guidelines/strobe/strobe-checklist.docx
